# Genetic engineering of *Pyrococcus furiosus* to use chitin as a carbon source

**DOI:** 10.1186/1472-6750-13-9

**Published:** 2013-02-07

**Authors:** Martina Kreuzer, Karolin Schmutzler, Ingrid Waege, Michael Thomm, Winfried Hausner

**Affiliations:** 1 Lehrstuhl für Mikrobiologie und Archaeenzentrum, University of Regensburg, Regensburg, 93053, Germany

## Abstract

**Background:**

Bioinformatic analysis of the genes coding for the chitinase in *Pyrococcus furiosus* and *Thermococcus kodakarensis* revealed that most likely a one nucleotide insertion in *Pyrococcus* caused a frame shift in the chitinase gene. This splits the enzyme into two separate genes, PF1233 and PF1234, in comparison to *Thermococcus kodakarensis*. Furthermore, our attempts to grow the wild type strain of *Pyrococcus furiosus* on chitin were negative. From these data we assume that *Pyrococcus furiosus* is most likely unable to use chitin as a carbon source. The aim of this study was to analyze *in vivo* if the one nucleotide insertion is responsible for the inability to grow on chitin, using a recently described genetic system for *Pyrococcus furiosus*.

**Results:**

A marker-less genetic system for *Pyrococcus furiosus* was developed using simvastatin for positive selection and 6-methylpurine for negative selection. Resistance against simvastatin was achieved by overexpression of the hydroxymethylglutaryl coenzyme A reductase gene. For the resistance to 6-methylpurine the hypoxanthine-guanine phosphoribosyltransferase gene was deleted. This system was used to delete the additional nucleotide at position 1006 in PF1234. The resulting chitinase in the mutant strain was a single subunit enzyme and aligns perfectly to the enzyme from *Thermococcus kodakarensis*. A detailed analysis of the wild type and the mutant using counted cell numbers as well as ATP and acetate production as growth indicators revealed that only the mutant is able to use chitin as a carbon source. An additional mutant strain containing a reduced chitinase version containing just one catalytic and one chitin-binding domain showed diminished growth on chitin in comparison to the mutant containing the single large enzyme.

**Conclusions:**

Wild type *Pyrococcus furiosus* is most likely unable to grow on chitin in the natural biotope due to a nucleotide insertion which separates the chitinase gene into two ORFs, whereas a genetically engineered strain with the deleted nucleotide is able to grow on chitin. The overall high sequence identity of the two chitinases between *P. furiosus* and *T. kodakarensis* indicates that this mutation occurred very recently or there is still some kind of selection pressure for a functional enzyme using programmed +/−1 frameshifting.

## Background

Chitin is the second most abundant polysaccharide on earth after cellulose. It is the major component of the exoskeletons of insects, the shells of crustaceans and of fungal cell walls [1]. Chitin consists of N-acetylglucosamine subunits which are linked by β-1,4-glycosidic bonds. The degradation of chitin is catalyzed by chitinases which hydrolyze these β-1,4-glycosidic bonds. Based on amino acid sequence similarity, chitinases have been classified into the glycoside hydrolases families 18 and 19 [2]. Family 18 chitinases contain a multidomain structure and are widely distributed in all domains of life. The common features of these enzymes are catalytic domains which consists of a (βα)_8_ (TIM barrel) fold with a conserved DXDXE motif and chitin-binding domains (ChBD) which are involved in the binding to the substrate [3,4].

Most chitinases described so far have been found in the eukaryal and the bacterial domains [5]. Within the domain of *Archaea*, only ten euryarchaeal chitinases and one crenarchaeal have been identified so far [6]. Most of these archaeal enzymes have been only annotated by sequence comparison. Experimental data about the activity and the structure of the enzymes are limited to the genera of *Halobacterium*, *Thermococcus* and *Pyrococcus*[7-10]. The first and best characterized archaeal chitinase was from *Thermococcus kodakarensis*[7,11]. A mutational analysis revealed that the enzyme possesses two catalytic domains (A and B) and three ChBDs [7]. The N-terminal catalytic domain A functions as an exochitinase and liberates diacetyl-chitobiose. The C-terminal catalytic domain B acts as an endochitinase which produces N-acetyl-chitooligosaccharides of various length, which could be further hydrolyzed to diacetyl-chitobiose by the N-terminal catalytic domain A. Further degradation to N-acetylglucosamine is the result of a concerted action of diacetyl-chitobiose deacetylase and exo-β-D-glucosaminidase [12].

In contrast to the single chitinase of *Thermococcus kodakarensis* the chitin-degrading enzymes of *Pyrococcus furiosus* are encoded by two open reading frames ChiA (PF1234) and ChiB (PF1233) which are separated by 37 nucleotides [8]. The gene product of PF1233 has a ChBD and a catalytic domain which is closely related to the *T. kodakarensis* catalytic domain B. The structure of this catalytic domain was determined in detail by NMR and X-ray analysis [13-15]. These data also indicate an endochitinase activity for PF1233 very similar to *T. kodakarensis*, however in contrast to the extracellular *T. kodakarensis* enzyme, the separated *P. furiosus* enzyme has no signal peptide at the N-terminal region. This would mean that the *P. furiosus* enzyme is intracellular and the substrates have to be imported into the cell.

A few years ago, Oku and Ishikawa suggested a different explanation for this observation: In principle, *P. furiosus* has also a single chitinase gene like *T. kodakarensis*, but a one nucleotide insertion at position 1006 in PF1234 caused a frame shift which resulted in the separation of the chitinase gene into two genes. Furthermore, their attempts to grow *P. furiosus* on chitin failed [16]. This result is in perfect agreement with previous growth experiments on chitin which also failed [17,18]. So far, there is only one report that *P. furiosus* is able to use chitin as a carbon source [8].

To reveal this issue, we used a genetic system for *P. furiosus* which allows the removal of the one nucleotide insertion in PF1234 to redesign the chitinase to a single enzyme [19]. Growth experiments with the wildtype and the mutant clearly demonstrate that the wild type strain of *P. furiosus* is - in contrast to the mutant with the redesigned chitinase - unable to efficiently use chitin as the main carbon source.

## Methods

### Strains and growth conditions

*P. furiosus* was cultivated at 90°C in SME medium, as described previously [20]. For the growth of *Pyrococcus* strain MUR27Pf with the deleted *xanthine-guanine phosphoribosyltransferase* gene (*xgprt*) the medium was supplemented with 6 mM guanosine monophosphate (Sigma, St. Louis, USA). For solidification, gelrite was added to a final concentration of 1%. The antibiotic simvastatin (Toronto Research Inc., Toronto, Canada) was dissolved in ethanol and 6-methylpurine (Sigma, St. Louis, USA) was dissolved in water. Both supplements were sterilized by filtration. SME-chitin medium was supplemented with 0.5% colloidal chitin, 0.025% yeast extract and 0.025% peptone.

For preparation of colloidal chitin 20 g of chitin powder (practical grade, from shrimp shells; Sigma, St. Louis, USA) were mixed with at least 200 ml 37% HCl (pre-cooled to 4°C) and stirred for 1 h at 4°C [21]. The suspension was poured into 1 l of H_2_O (pre-cooled to 4°C) and was filtered through paper filter (311853, Schleicher and Schüll, Dassel, Germany). The filtrate was washed three times with 1 l of H_2_O, resuspended in 1 l of H_2_O and neutralized by the addition of NaOH until pH 7.0. The suspension was filtered and washed with 3 l of H_2_O to deionize the chitin. The resulting suspension was filtered and a part was dried to determine the content of liquidity.

*Escherichia coli* strain DH5α was used as a host strain for plasmid constructions and was cultivated at 37°C in Luria-Bertani (LB) medium. For the selection of transformants, ampicillin was added at 100 μg ml^-1^ to the medium.

### General DNA manipulations and plasmid constructions

Restriction enzymes and DNA polymerases for PCR reactions were purchased from NEB (Ipswich, USA). Plasmid DNA and DNA fragments from agarose gels were isolated using a Wizard® Plus SV Miniprep DNA Purification System or Wizard® SV Gel and PCR Clean-Up System from Promega (Mannheim, Germany). DNA sequencing was performed by Seqlab (Göttingen, Germany). Genomic DNA from *P. furiosus* wild type and genectically engineered strains was isolated as described previously [19].

The plasmid pMUR37 was created to enable a markerless deletion of PF1950 (*xgprt*). It contained three DNA fragments which were joined by single overlap extension PCR reactions: An upstream region of PF1950 (primers Pf1950single_mi_fus-F 5´ -aacagaagtttaagccttcgaagaattgggaagagggaga-3´ and Pf1950ml_up_fus1500bp_R 5´ -gctttttccttatccactacttatatgaccgcaggtattc-3´ ), a downstream region of PF1950 (primers Pf1950ml_mi-fus1500bp_F 5´ -gaatacctgcggtcatataagtagtggataaggaaaaagc-3´ and Pf1950_hr_do_R 5´ - gttgaaacagttgcaactcttgg-3´ ) and for the selection with simvastatin the resistance cassette (primers Pf1950single_up_BamHI_F 5´ -gggcccggatccgggcatttcatcattttt-3´ and Pf1950single_up_fus_R 5´ -tctccctcttcccaattcttcgaaggcttaaacttctgtt-3´ ) as described previously [19]. The fused fragment was hydrolyzed with BamHI and SacI and was ligated into the corresponding sites of pUC19.

For the construction of the plasmids pMUR47 and pMUR50, a modified pUC19 plasmid with an additional AscI recognition sequence within the multiple cloning site was used. For both constructs two PCR fusion products were ligated using a common NotI restriction site and inserted into the modified vector using AscI and SbfI restriction sites.

In the case of pMUR47 the first fusion PCR product with the deleted nucleotide was created using the following two primer pairs: (Pf1234_AscI_F 5´ -atcgaaggcgcgcctgctcggtattgtgcttgc-3´ /Pf1234_Del_Fus_R 5´ -tttatcttctaattcggcttgatc-3´ ) and (Pf1233_Del_Fus_F 5´ -ataaaaaagagtatctcctaactgcagc-3´ / Pf1233_NotI_A_R 5´ -ggtgcagcggccgctggagttggtgatggtgttg-3´). The second fusion PCR product consisted of a two-gene resistance cassette which was needed for the selection-counter-selection system. The resistance cassette contained a *gdh* promoter, the *hmgCoA* reductase from *T. kodakarensis*, the region coding for the *xgprt* (PF1950) and the histone A1 terminator sequence of *P. furiosus*[19]. The first part was amplified using the primers SimV_NotI_F 5´ -gatgcgcggccgcgggcatttcatcatttttatgaactttgatgaacg-3´ and SimV_Rv 5´ -tcaccctagaaaaagataagcc-3´ . For the second part, the primer pair Pf1950_F_fus1233A 5´ -gcttatctttttctagggtgacctgggatccaattaccg-3´ and Pf1233_SbfI_R 5´ -atacggcctgcaggttggagtgggtgtggg-3´ was used. Both PCR products were combined with single-overlap extension PCR.

Plasmid pMUR50 was constructed by combination of a PCR product containing the upstream region up to the signal peptide region of PF1234 (primers Pfup1234_AscI_B_F 5´ -atacgaggcgcgccaactccaatttccctgagc-3´ and Pf SP_up1234_ R 5´ -ggccgatactggatagaatagagatat-3´ ) and a PCR product coding for the catalytic domain of PF1233 (primers Pf SP_1233_fus_F5´ -tatccagtatcggccactacccctgtcccag-3´ and Pf1233_NotI_B_R 5´ -cctaatgcggccgctagaggaattgagcctgc-3´ ). The resulting PCR product was combined with the PCR-amplified resistance cassette using primer pair Pf1950_NotI_F 5´ -tagcatgcggccgctcaccctagaaaaagataagcc-3´ and PfhmgCoA_SbfIR 5´ -gataggcctgcagggggcatttcatcatttttatg-3´ and inserted into the modified vector as mentioned before. The resulting constructs were verified by DNA sequencing.

### Transformation of *P. furiosus*

Standard heat shock transformation of *P. furiosus* was performed as described previously [19]. To obtain the marker-less mutant MUR27Pf, circular plasmid DNA of pMUR37 was used and the corresponding transformants were selected with 10 μM simvastatin in SME-starch liquid medium at 85°C for 48 h. Pure cultures of the intermediate mutant MUR27Pf_i were obtained by plating the cells on solidified medium in the presence of 10 μM simvastatin. The integration of the plasmid into the genome by single cross-over was verified by analyzing corresponding PCR products.

Cultures of the correct intermediate mutant were washed with medium under anaerobic conditions to remove the simvastatin. In detail, 1.5 ml of a grown culture were centrifuged in an anaerobic chamber for 4 min at 6,000g and resuspended in fresh culture medium without simvastatin. This procedure was repeated three times. For the counter selection the cultures were plated in the presence of 50 μM 6-methylpurine to induce a second homologous recombination step to recycle the selection marker and to eliminate integrated plasmid sequences. In the case of mutant MUR23Pf linearized plasmid pMUR47 was used for the transformation. The genotype of the final mutants was confirmed by PCR and Southern blot experiments.

### Growth analysis on chitin medium

For a more detailed analysis of the growth behavior of *Pyrococcus* on chitin, bottles with 200 ml chitin medium were used and incubated at 90°C. Cell numbers were analyzed with a Thoma counting chamber (0.02-mm depth; Marienfeld, Lauda-Königshofen, Germany) using a phase-contrast microscope.

For the preparation of cell extracts to measure the ATP content, 0.5 ml of *Pyrococcus* cultures was centrifuged (3 minutes, 10,000g). The cell pellet was washed three times in 0.8 ml PBS buffer, resuspended in 200 μl PBS and treated with glass beads using a FastPrep-24 (M. P. Biomedicals, Irvine, CA) for cell lysis. After centrifugation (10,000g for 3 minutes at 4°C), the ATP amount in the supernatants was quantified by a luciferin/luciferase assay (FluoProbes, Interchim, Montluçon Cedex, France) using a portable tube luminometer (Junior LB 9509, Berthold Technologies, Bad Wildbad, Germany) according to the instruction manual.

The amount of acetate in the culture supernatant was analyzed using an enzymatic acetate determination kit (R-biopharm, Darmstadt, Germany). For quantification 0.5 ml of a *Pyrococcus* culture was centrifuged and used as indicated in the operating guidelines.

## Results and discussion

**Figure 1:**
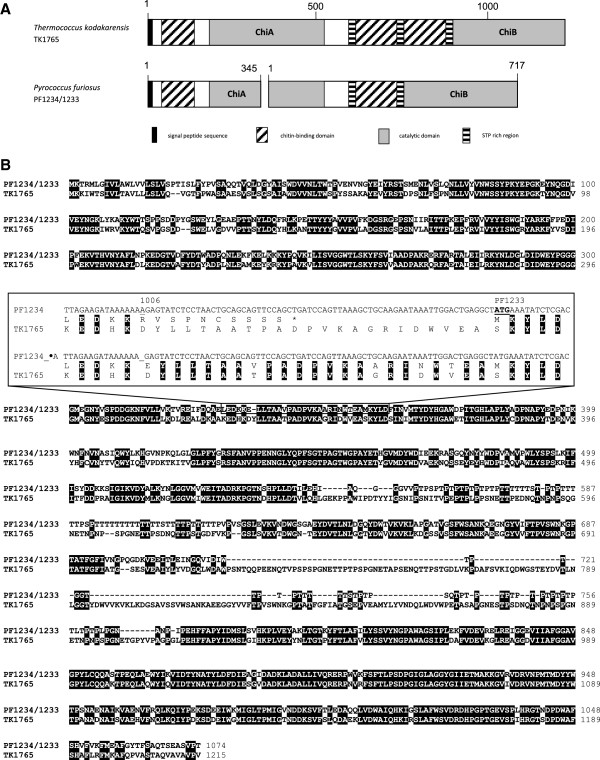
**Comparison of chitinases from *P. furiosus *and *T. kodakarensis.*** (**A**) Schematic models of catalytic and chitin binding domains of both chitinases, the single enzyme from *T. kodakarensis* and the split version from *P. furiosus*. (**B**) Alignment of the amino acid sequences. In the case of *P. furiosus* the corresponding DNA sequence was modified by the deletion of the adenine at position 1006. The details of this region were shown in the box within the alignment. The upper part within the box shows the original DNA sequence of *P. furiosus* together with the corresponding amino acid sequence in the region of the putative nucleotide insertion of PF1234. The lower part shows the sequence with the deleted nucleotide. For sequence comparison, the amino acid sequence of the corresponding chitinase region of *T. kodakarensis* is also shown and identical amino acids between both, *T. kodakarensis* and *P. furiosus*, are shadowed in black. The position with the additional nucleotide is underlined. The putative stop codon is marked by an asterisk.

A detailed sequence analysis of the *P. furiosus* chitinase genes around the gene split of PF1234 and PF1233 confirmed the suggestion of Oku and Ishikawa [16] that a one nucleotide insertion at position 1006 in PF1234 caused a frame shift which resulted in the generation of a stop codon after a stretch of ten amino acids (Figure 1). The second chitinase gene PF1233 starts 37 bp downstream of PF1234. RT-PCR experiments indicate that both genes were separately transcribed for growth on chitin as well as on tryptone-containing medium lacking chitin [8]. Nevertheless, it is difficult to localize a strong promoter signal upstream of PF1233. Furthermore, a detailed comparison with the corresponding region of the single chitinase gene from *T. kodakarensis* clearly indicates that the deletion of an adenine residue at position 1006 allows a much better sequence alignment between *P. furiosus* and *T. kodakarensis* within this region (Figure 1). 

To exclude the possibilities that the presence of this additional nucleotide is caused by a sequencing error or is only present in the *P. furiosus* strain used for the genome sequencing project [22] we ordered a new *P. furiosus* strain (DSM 3638) from the DSMZ and confirmed the presence of this additional nucleotide by sequencing of a corresponding PCR product (data not shown).

*In vitro* experiments using an artificial recombinant chitinase from *P. furiosus*, heterologously expressed in *E. coli*, indicate that the single enzyme constructed by the deletion of the additional nucleotide is much more active than the separated wild type enzymes [16]. To investigate if this finding from the *in vitro* experiments could be confirmed by *in vivo* data we used a modified version of the recently developed genetic system for *P. furiosus* to delete this additional nucleotide in the genome [19].

As the genetic system described so far is based on a shuttle vector and did not allow the modification of genes within the genome we started the genetic modification of *P. furiosus* with the establishment of a selection/counter selection (pop-in/pop-out) system according to a genetic system which was recently described for *T. kodakarensis*[23,24]. Santangelo et al. demonstrated that the deletion of the *xgprt* gene (TK0664) confers resistance to 6-methylpurine. First experiments to inactivate the corresponding gene PF1950 in *P. furiosus* indicated that this gene can be also used for counter selection experiments in *P. furiosus* (data not shown).

**Figure 2:**
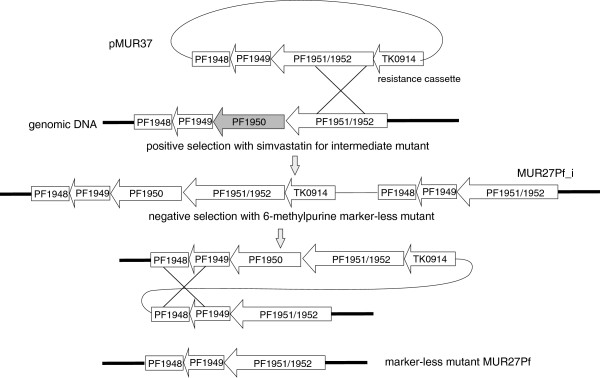
**Schematic drawing for the construction of marker**-**less strain MUR27Pf.** Plasmid pMUR37 was obtained by overlapping PCR. It contained 1-kb upstream and downstream regions of PF1950 and the resistance cassette TK0914 for selection with the antibiotics simvastatin. After transformation the intermediate mutant MUR27Pf_i was isolated and characterized. An additional negative selection with 6-methylpurine produced strain MUR27Pf with the marker-less deletion of PF1950 (shadowed in grey).

To establish a deletion mutant for PF1950 in *P. furiosus*, plasmid pMUR37 was constructed (Figure 2). This plasmid contains about 400 bp upstream and 1500 bp downstream sequence of PF1950 followed by the *hydroxymethylglutaryl coenzyme A* (*hmg-CoA*) *reductase* gene from *T. kodakarensis* (TK0914). The expression of this gene under the control of a strong *gdh* promoter conveys resistance to the antibiotics simvastatin [19,25]. The resulting plasmid pMUR37 was integrated into the wild type *P. furiosus* by single cross-over into the homologous genomic region of PF1951/PF1952 by transformation (Figure 2). A successful intermediate mutant (MUR27Pf_i) was selected based on the resistance toward simvastatin and further characterized by PCR analysis (data not shown). For the marker-less deletion of PF1950 a negative selection with 6-methylpurine was performed to induce a second recombination event which enabled the removal of the complete plasmid together with the resistance cassette (pop-out mechanism). The resulting *P. furiosus* mutant with the deletion of PF1950 (MUR27Pf) was verified by PCR and Southern blot experiments (data not shown). Further characterization of MUR27Pf revealed that the presence of 6 mM guanine monophosphate in the medium improved the growth of this strain (data not shown). Furthermore, in combination with the positive simvastatin selection marker the new strain enables marker-less gene modifications within the genome of *P. furiosus*. 

**Figure 3:**
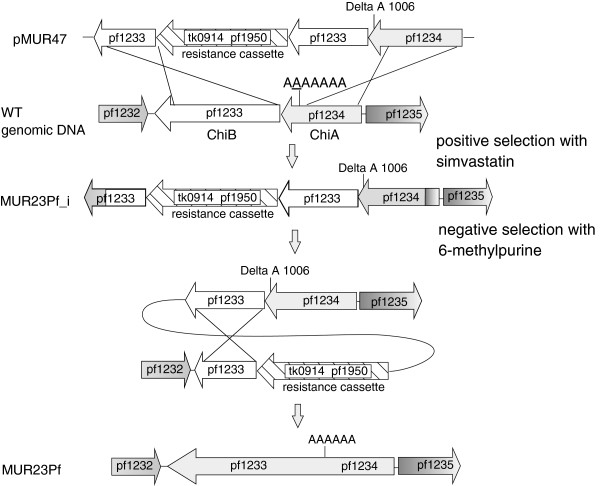
**Cloning strategy for the mutant MUR23Pf.** Plasmid pMUR47 was constructed by overlapping PCR. It contained the chitinase sequence with the deleted nucleotide at position 1006 with corresponding upstream and downstream regions for homologous recombination together with the resistance cassette consisting of TK0914 for selection with the antibiotics simvastatin and PF1950 for the counter selection with 6-methylpurine. After transformation with linearized pMUR47 the intermediate mutant MUR23Pf_i was isolated and characterized. The negative selection with 6-methylpurine resulted in strain MUR23Pf with the redesigned chitinase.

In the next step we used the new strain MUR27Pf for the redesign of the chitinase to a single enzyme. For the deletion of the additional adenine nucleotide at position 1006 within PF1234 plasmid pMUR47 was constructed. This plasmid contains a two-gene resistance cassette consisting of the *hmg-CoA reductase* from *T. kodakarensis* (TK0914) and the *xgprt* (PF1950) from *P. furiosus* (Figure 3). Both genes were transcribed together under the control of the *gdh* promoter of *T. kodakarensis* and the terminator of the histone *A1* gene [19]. This fragment was fused by single-overlap extension PCR with a part of PF1233 on one side and with the modified region of PF1233/PF1234 containing the sequence with the deleted nucleotide at position 1006 on the other side. After cloning in *E. coli* and sequence verification the resulting construct pMUR47 was hydrolyzed with restriction enyzmes to remove the vector DNA and used for transformation of *P. furiosus* by homologous recombination via double cross-over (Figure 3). Transformants with the integrated DNA fragment were selected with the antibiotic simvastatin. After characterization of the intermediate mutant a negative selection with 6-methylpurine was performed to create the marker-less *P. furiosus* MUR23Pf strain with the single chitinase gene. 

**Figure 4:**
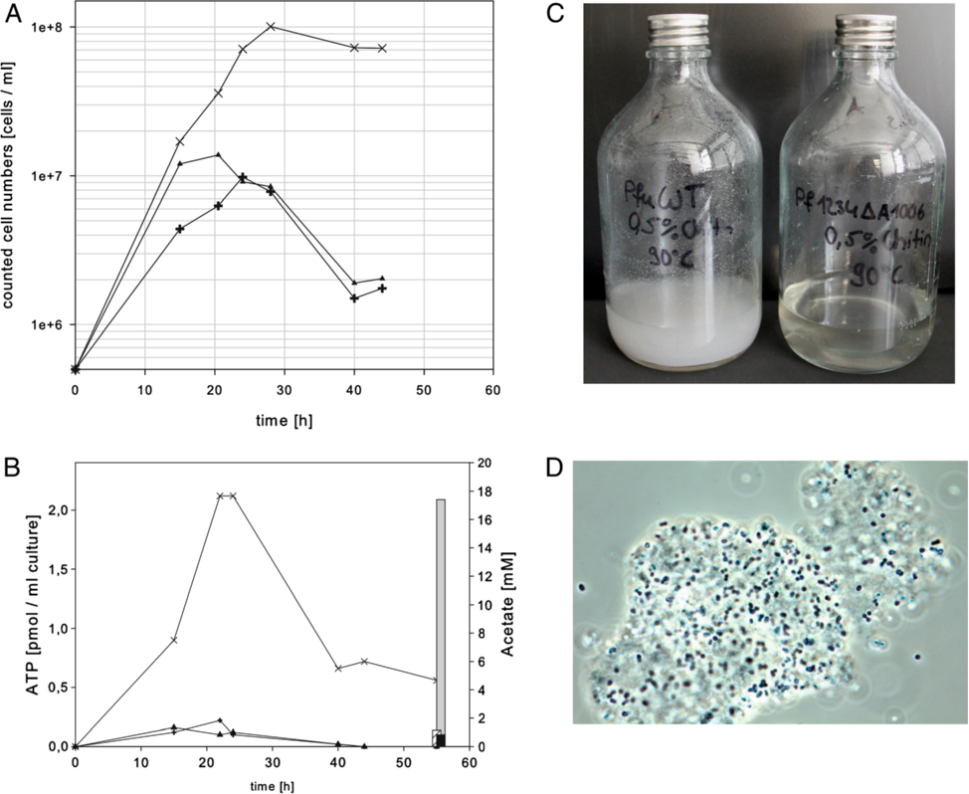
**Growth behavior in SME medium with 0.5% colloidal chitin at 90°C.** (**A**) Growth curves of wild type *P. furiosus* (plus signs), mutant MUR27Pf (triangles) and mutant MUR23Pf (crossings) with the redesigned chitinase as a single enzyme. (**B**) Growth analysis of the *Pyrococcus* strains by quantification of the ATP level (left axis). In addition, the final acetate concentration in the medium was analyzed after 55 h incubation (right axis). (**C**) Documentation of the incubated bottles after x h incubation, *P. furiosus* wild type is shown on the left side and mutant MUR23Pf on the right side. (**D**) Phase contrast microscopic picture of the mutant strain MUR23Pf grown on chitin.

After verification of the resulting mutant MUR23Pf by PCR and Southern blot experiments (data not shown), the mutant strains, MUR23Pf and MUR27Pf, and the wild type of *P. furiosus* were grown in SME medium in the presence of 0.5% colloidal chitin, 0.025% yeast extract and 0.025% peptone. The mutant with the single chitinase reached a maximum cell density of 1 × 10^8^ ml^-1^ after approximately 28 h and remained stable in the stationary phase for at least additional 20 h (Figure 4A). In contrast, the wild type and the strain with the deletion of the *xgprt* gene reached a ten-fold lower cell density of about 1 × 10^7^ ml^-1^ after 28 h and further incubation resulted in a strong decrease in the cell density (Figure 4A). The quantification of the ATP level revealed that the ATP concentration of the mutant with the redesigned chitinase was also about 10-fold higher than the ATP concentration of the control strains (Figure 4B). The determination of the acetate concentration after 55 h incubation indicated a strong increase almost up to 18 mM acetate in the medium of the strain with the modified chitinase, whereas the media of the control strains exhibited acetate concentrations below 1 mM (Figure 4B).

This strong difference in the observed acetate concentrations clearly indicates that the genetically engineered *P. furiosus* strain uses chitin as a carbon source. Chitin is highly acetylated and we therefore assume that acetate is released from the chitin during growth of *P. furiosus*. In comparison, in an experiment with similar cell density and incubation time grown on cellobiose as substrate, an acetate concentration of about 4 mM was observed [26]. Due to the insolubility of the chitin and the fact that many cells stick on the chitin it was very difficult to report reliable OD values, but the visual inspection of the incubated bottles of the wild type and the mutant strain clearly demonstrated chitin degradation (Figure 4C). The bottle containing the wild type after an incubation of 55 h still exhibited a milky turbidity due to the insolubility of the chitin. In contrast, the amount of insoluble chitin in the bottle with the genetically engineered *P. furiosus* strain was considerably reduced (Figure 4C). Figure 4D shows a phase contrast microscopic picture of the mutant strain. It can be clearly seen that the cells are attached to the chitin particles.

The experiments presented so far clearly indicate that the *P. furiosus* strain with the redesigned chitinase can grow much better on colloidal chitin than the wild type strain. In this context it is interesting to note the overall high sequence identity of the two chitinases between *P. furiosus* and *T. kodakarensis*. This indicates that this mutation occurred very recently or there is still some kind of selection pressure for a functional enzyme. It is possible that *P. furiosus* is able to synthesize the chitinase as a single enzyme using programmed +/−1 frameshifting which enables the ribosome to bypass the frameshift [27]. This situation was recently described for the expression of the *fucA1* gene in *Sulfolobus acidocaldarius*. However, in this case the expression efficiency is only about 5% in comparison to a gene without the programmed frameshift [27]. To exclude or to confirm the idea of programmed frameshifting additional experiments will be necessary. It is not possible to use available proteomics data of *P. furiosus* to support this idea as the available proteomics data do not match the chitinases. Furthermore, first attempts to use purified enzyme for a detailed mass spectrometry-based analysis were complicated by the finding that the enzyme is most likely the target of intensive proteolytic cleavage activity (data not shown).

**Figure 5:**
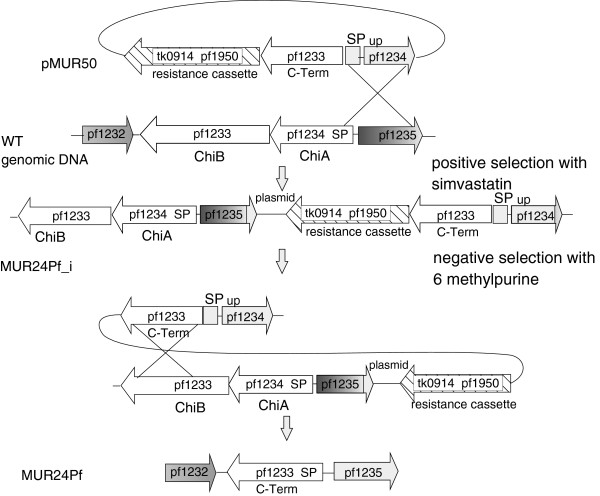
**Schematic drawing for the construction of strain MUR24Pf.** Plasmid pMUR50 possesses a modified chitinase construct together with the resistance cassette as used for the construction of plasmid pMUR47. After transformation with circular pMUR50 the intermediate mutant MUR24Pf_i was isolated and characterized. The negative selection with 6-methylpurine resulted in strain MUR24Pf with a modified version of the chitinase.

To analyze if we could further stimulate the growth of *P. furiosus* on chitin we constructed an additional mutant. Oku and Ishikawa also demonstrated in *in vitro* experiments that a reduced recombinant version of the chitinase consisting of the chitin binding and the catalytic domain B had a higher activity as the whole enzyme [16]. To analyze if this *in vitro* result with heterologous expressed proteins could be confirmed *in vivo*, the additional plasmid pMUR50 was created (Figure 5). It contained the resistance cassette, the catalytic and the chitin binding domain of PF1233, the signal peptide sequence of PF1234 and the corresponding upstream sequence for homologous recombination by single cross-over. After sequence verification the plasmid was used to create the marker-less *P. furiosus* mutant MUR24Pf using the same strategy as described before (Figure 5). 

**Figure 6:**
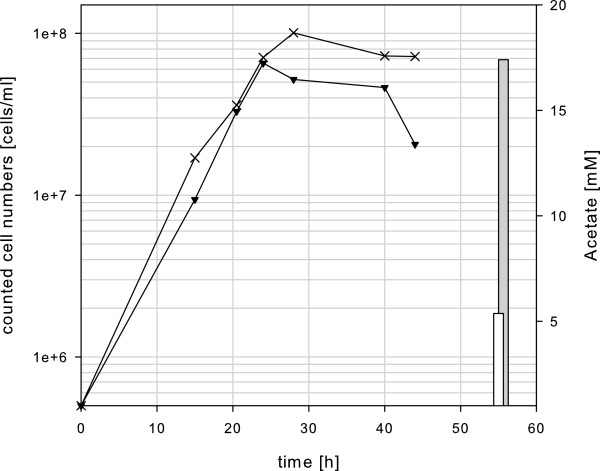
**Growth curves (left axis) and final acetate concentrations (right axis) of the *Pyrococcus *strains MUR23Pf (crossings) and MUR24Pf (triangles).**

We also performed growth experiments using similar conditions as outlined above with the strain containing the ChiB domain (Figure 6). For comparison, the data of the mutant strain MUR23Pf with the redesigned chitinase as a single enzyme were included. The maximum cell density of the strain with the ChiB domain was reduced from 1 × 10^8^ ml^-1^ to 6.5 × 10^7^ ml^-1^. Furthermore, the stability of the cells in the stationary phase was lower and the acetate concentration in the medium was reduced by one third in comparison to the strain with the redesigned complete chitinase (Figure 6). Taken together, the data clearly indicate that the *P. furiosus* strain with the redesigned complete chitinase as a single enzyme had a growth advantage compared to the strain with the smaller version containing the ChiB domain. This result did not confirm the *in vitro* data from Oku and Ishikawa and is therefore a nice example that sometimes *in vitro* results do not match with *in vivo* results [16]. 

## Conclusions

Our data demonstrate that wild type *P. furiosus* is most likely unable to use chitin as a main carbon source due to a one nucleotide insertion which splits the chitinase into two separate enzymes. In contrast, a genetically engineered strain with the deleted nucleotide is able to grow on chitin. The overall high sequence identity of the two chitinases between *P. furiosus* and *T. kodakarensis* indicates that this mutation occurred very recently or there is still some kind of selection pressure for a functional enzyme using programmed +/−1 frameshifting [27]. As the later one resulted in a very low expression level of the chitinase, we conclude that *P. furiosus* does not rely on chitin as a major carbon source in the natural biotope.

## Abbreviations

ChBD: Chitin binding domain; *fucA1*: α-fucosidase; *gdh*: Glutamate dehydrogenase; *hmg-CoA*: Hydroxymethylglutaryl-Coenyzme A; *P*: *Pyrococcus*; PCR: Polymerase chain reaction; *T*: *Thermococcus*; *Xgprt*: Hypoxanthine-guanine phosphoribosyltransferase.

## Competing interests

The authors declare that they have no competing interests.

## Authors’ contributions

MK participated in designing the data and carried out the experimental work on the chitinase mutants. KS established the marker-less system. IW was involved to set up the experimental work. WH and MT coordinated and supervised the research and wrote the manuscript. All authors read and approved the final manuscript.
